# Less is more? Assessing the validity of the ICD-11 model of PTSD across multiple trauma samples

**DOI:** 10.3402/ejpt.v6.28766

**Published:** 2015-10-07

**Authors:** Maj Hansen, Philip Hyland, Cherie Armour, Mark Shevlin, Ask Elklit

**Affiliations:** 1 Department of Psychology, National Centre for Psychotraumatology, University of Southern Denmark, Odense M, Denmark; 2 School of Business, National College of Ireland, Dublin, Ireland; 3 School of Psychology, Ulster University, Coleraine, UK

**Keywords:** CFA, PTSD, DSM-5, ICD-11, latent structure

## Abstract

**Background:**

In the 5th edition of the Diagnostic and Statistical Manual of Mental Disorders (DSM-5), the symptom profile of posttraumatic stress disorder (PTSD) was expanded to include 20 symptoms. An alternative model of PTSD is outlined in the proposed 11th edition of the International Classification of Diseases (ICD-11) that includes just six symptoms.

**Objectives and method:**

The objectives of the current study are: 1) to independently investigate the fit of the ICD-11 model of PTSD, and three DSM-5-based models of PTSD, across seven different trauma samples (*N*=3,746) using confirmatory factor analysis; 2) to assess the concurrent validity of the ICD-11 model of PTSD; and 3) to determine if there are significant differences in diagnostic rates between the ICD-11 guidelines and the DSM-5 criteria.

**Results:**

The ICD-11 model of PTSD was found to provide excellent model fit in six of the seven trauma samples, and tests of factorial invariance showed that the model performs equally well for males and females. DSM-5 models provided poor fit of the data. Concurrent validity was established as the ICD-11 PTSD factors were all moderately to strongly correlated with scores of depression, anxiety, dissociation, and aggression. Levels of association were similar for ICD-11 and DSM-5 suggesting that explanatory power is not affected due to the limited number of items included in the ICD-11 model. Diagnostic rates were significantly lower according to ICD-11 guidelines compared to the DSM-5 criteria.

**Conclusions:**

The proposed factor structure of the ICD-11 model of PTSD appears valid across multiple trauma types, possesses good concurrent validity, and is more stringent in terms of diagnosis compared to the DSM-5 criteria.

The field of trauma research and clinical practice is soon to experience a difficult problem; researchers and clinicians will be faced with a situation where the two standard diagnostic nomenclatures will provide considerably different descriptions of the same disorder. After two decades of research undermining the factorial validity of the three-factor model of posttraumatic stress disorder (PTSD) (e.g., Biehn et al., 2013; Yufik & Simms, 2010) set out in the 4th edition of the Diagnostic and Statistical Manual of Mental Disorders (DSM; American Psychiatric Association [APA], 1994), both the APA and the World Health Organization (WHO) independently sought to revise the description of this disorder.

For DSM-5 (APA, 2013), several symptoms were revised and three new symptoms were introduced, bringing the total number of symptoms to 20. The DSM-5 diagnosis is now a four-factor structure of intrusions (B1–B5), avoidance (C1 and C2), negative alternations in cognitions and mood (NACM: D1–D7), and alternations in arousal and reactivity (E1–E6). In the WHO's 11th revision of the International Classification of Diseases (ICD-11; Maercker et al., 2013), set for release in 2017, the goal was not to expand the symptom profile, but rather to substantially reduce the number of symptoms so that only specific symptom indicators of PTSD would be retained (Brewin, 2013). The proposed ICD-11 model of PTSD includes six symptoms belonging to three clusters; re-experiencing of the traumatic event(s) in the present accompanied by emotions of fear or horror; avoidance of traumatic reminders; and a sense of current threat that is manifested by excessive hypervigilance or an enhanced startle reaction (Maercker et al., 2013).

Since the release of DSM-5, several studies have investigated the latent structure of PTSD symptoms by comparing the DSM-5 four-factor model to alternative conceptualizations. Many of these studies have supported the DSM-5 model (Armour, Contractor, Palmieri, & Elhai, 2014; Biehn et al., 2013; Contractor et al., 2014; Elhai et al., 2012). However, as was the case with the DSM-IV model of PTSD, studies have begun to support alternative symptom structures to that which is outlined in the DSM-5. One study (Miller et al., 2013) supported a “Dysphoria model” which was defined by a broad dysphoria factor (criteria D and E except for symptoms of hypervigilance and exaggerated startle). In a later study, Forbes, Lockwood, Elhai, et al. (2015) were unable to distinguish between the dysphoria model and the DSM-5 model as both models fit the data equally well. Liu et al. (2014) tested competing models of the latent structure of PTSD and found support for a six-factor “Anhedonia model.” This model builds on the dysphoric arousal model (Elhai et al., 2011) by separating the NACM cluster into two factors based on theoretical and empirical studies showing that negative affect and positive affect are distinct constructs (Watson, 2009).

In contrast to the numerous studies testing the DSM-5's latent structure of PTSD, the latent structure of the proposed ICD-11 model of PTSD has received less empirical attention. Forbes, Lockwood, Creamer, et al. (2015) assessed 613 survivors of physical injury 6 years posttrauma and reported good model fit and good predictive validity through assessments of disability and poor psychological quality of life. Tay, Rees, Chen, Kareth, and Silove (2015) assessed the fit of the ICD-11 model in a sample of West Papuan refugees and found excellent model fit. In this study, the authors also assessed the fit of the DSM-5 model of PTSD and reported similar model fit results for both systems.

Several studies across multiple trauma samples using latent class/profile analysis have provided additional support for the ICD-11 proposals (Cloitre, Gavert, Brewin, Bryant, & Maercker, 2013; Cloitre, Gavert, Weiss, Carlson, & Bryant, 2014; Elklit, Hyland, & Shevlin, 2014b). Results from these studies found distinct classes reflecting those suffering from PTSD and complex-PTSD, as per ICD-11 guidelines. Moreover, participants suffering from PTSD exhibited substantially greater psychological distress compared to those without PTSD.

Although the DSM-5 and ICD-11 have diverged substantially with respect to the number of symptom indicators of PTSD, the two systems have harmonized with respect to the diagnostic features. Unlike the ICD-10, and congruent with the DSM-5, the ICD-11 now includes a requirement of functional impairment, and describes PTSD as a disorder that arises shortly after traumatic exposure which must persist for several weeks. The harmonization with respect to diagnostic features of PTSD has led researchers to compare prevalence rates between the DSM (IV and 5) and ICD-11. Generally, results have suggested little difference in prevalence estimates between the two diagnostic systems. Two studies compared diagnostic rates between ICD-11 and DSM-IV and found no differences (Morina, Van Emmerik, Andrews, & Brewin, 2014; Van Emmerik & Kamphuis, 2011). Additionally, two studies have compared DSM-5 prevalence rates to ICD-11 prevalence rates with one study revealing that the DSM-5 produces higher diagnostic rates (6.7 vs. 3.3%; O'Donnell et al., 2014), and another suggesting broadly similar diagnostic rates (ICD-11=3.2% vs. DSM-5=3.0%; Stein et al., 2014).

The presence of two widely discrepant methods of conceptualizing what is purported to be the same distressing psychological experience is highly problematic. Identifying an accurate symptom configuration of PTSD is imperative as it can be used to inform clinical understandings of the etiology and maintenance of the disorder (Elhai & Palmieri, 2011), whereas inaccurate diagnostic criteria can lead to functionally impaired individuals failing to receive necessary support (underdiagnosis), or alternatively, individuals who are displaying normal responses to trauma being wrongly diagnosed (overdiagnosis). The current study is carried out with a number of objectives in mind. First, in order to redress the lack of data assessing the construct validity of the ICD-11 model of PTSD, this study will assess the statistical fit of the model to data obtained from seven different trauma populations. Simultaneously, the fit of the DSM-5 model will be compared to alternative DSM-5-based models previously supported in the literature. It is critical to note that given the widely discrepant number of symptoms in the ICD-11 model (6), compared to the DSM-5 model (20), it is not possible to provide a direct empirical comparison of the two models using standard model comparison indices. This study will assess the fit of each model to the data obtained from the seven trauma samples in order to determine which model represents the data best. Second, this study will seek to determine the concurrent validity of the ICD-11 model of PTSD through assessments of association with a set of related psychological experiences (depression, anxiety, dissociation, and aggression). This will also determine if the explanatory power of the ICD-11 model of PTSD is reduced relative to the DSM-5 model. Third, diagnostic rates of PTSD based on the ICD-11 and DSM-5 criteria will be compared.

## Materials and method

### Participants and procedures

Data from a total of 3,746 participants were used for the current study. The mean age for the entire sample was 38.40 (SD=11.32, range 18–80) and the majority of respondents were female (71%). Participants were drawn from seven independent trauma samples. All studies were granted ethical approval from either the University of Aarhus or the University of Southern Denmark.

Sample 1 comprised bereaved parents who had suffered the death of a child (*N*=666). Most parents were members of the Danish “National Association of Infant Death” and experienced the loss of a child on average 3.3 years from the time of participating in the study. The mean age was 33.90 (SD=5.90, range 18–62) and 57% were females (Murphy, Shevlin, & Elklit, 2014).

Sample 2 comprised victims of road traffic accidents suffering from whiplash (*N*=1,664). The participants had been exposed to the trauma on average 62 months prior to participating in the study and were recruited through the “Danish Society for Polio, Traffic and Accident Victims.” The mean age of the sample was 42.96 (SD=10.21, range 20–77) and 79% of victims were female (Elklit et al., 2014b).

Sample 3 comprised sufferers of paraplegia (*N*=218). The participants were recruited from two Danish rehabilitation centers and the Danish Paraplegic Association 1 month to 53 years after their injury (*M=*14.0 years, SD*=*10.1 years). The mean age was 44.07 (SD=13.12, range 18–80) and the majority of participants (69%) were male (Nielsen, 2003).

Sample 4 comprised victims of a physical assault (*N*=191). Participants in this sample were recruited during a 1-month period from an emergency ward at the University Hospital of Aarhus after exposure to “grievous bodily harm caused by another person.” The majority of victims were male (72%) and the mean age was 31.92 (SD=11.67, 18–80) (Elklit et al., 2014b).

Sample 5 comprised victims of incest (*N*=503). The participants were recruited through the Danish incest support centers as adults. The mean age of this sample was 36.43 (SD*=*10.81, range 18–77) with the majority of victims being females (87%) (Elklit, Christiansen, Palic, Karsberg, & Eriksen, 2014a).

Sample 6 was a primarily female (98%) sample of sexual assault victims (*N*=293) assessed 3 months after the assault. The participants had all contacted the “Centre for Rape Victims” located within the University Hospital of Aarhus. The mean age of the sample was 22.46 (SD=9.11, range 18–70) (Shevlin, Hyland, & Elklit, 2014).

Sample 7 comprised a heterogeneous sample of trauma patients who were currently receiving psychological treatment (*N*=203). Participants were recruited through the crisis aid service, “Falck Health Care,” and questionnaires were completed approximately 7–10 days after the traumatic exposure (i.e., death, threats, harassment, and assault). The mean age of the sample was 37.61 (SD=12.07, range 18–77) and 66% were females (Elklit, 2000).

### Measures

The Harvard Trauma Questionnaire Part IV (HTQ; Mollica et al., 1992) includes 31 items designed to assess DSM-IV PTSD symptoms and more general posttraumatic stress symptoms. Answers are rated on a four-point Likert scale (1=*not at all*, to 4=*all the time*). Although designed to reflect the DSM-IV, the HTQ contains additional items that largely reflect the newly introduced PTSD symptoms in the DSM-5. The mapping of each HTQ item to the models of PTSD can be seen in Table 1. There are two limitations associated with using the HTQ to capture the DSM-5 PTSD symptoms: 1) one item is used to measure both physiological and psychological reactivity to reminders of the traumatic event (B4 and B5) and 2) there is no item that can assess the newly introduced symptom of reckless or self-destructive behavior (E2). The Danish version of the HTQ has been used in a wide range of trauma populations with reports of good reliability and validity (Bach, 2003). Cronbach's alpha (α) for the current study was high (*α*=0.91).

**Table 1:** Item mapping for the four PTSD models

DSM-5 symptoms of PTSD	HTQ items	Model 1 ICD-11	Model 2 DSM-5	Model 3 Dysphoric Arousal^a^	Model 4 Anhedonia^b^
B1. Intrusive thoughts	HTQ1. Recurrent thoughts or memories of the most hurtful or terrifying events	–	I	RE	RE
B2. Distressing dreams	HTQ3. Recurrent nightmares	RE	I	RE	RE
B3. Dissociative reactions	HTQ2. Feeling as though the event is happening again	RE	I	RE	RE
B4/5. Emotional reactivity and physiological reactivity	HTQ16. Sudden emotional or physical reaction when reminded of the most hurtful or traumatic events	–	I	RE	RE
C1. Efforts to avoid thoughts	HTQ15. Avoiding thought or feelings associated with the traumatic or hurtful events	A	A	A	A
C2. Efforts to avoid reminders	HTQ11. Avoiding activities that remind you of the traumatic or hurtful event	A	A	A	A
D1. Trauma-related amnesia	HTQ12. Inability to remember parts of the most hurtful or traumatic events	–	NACM	NACM	NACM
D2. Negative beliefs about oneself	HTQ14. Feeling as if you do not have a future	–	NACM	NACM	NACM
D3. Self-blame	HTQ19. Blaming yourself for the things that have happened	–	NACM	NACM	NACM
D4. Negative emotional state	HTQ23. Feeling ashamed of the hurtful or traumatic events that have happened to you HTQ21. Feeling guilty for having survived HTQ31. Feeling guilty for not doing anything or not doing enough	–	NACM	NACM	NACM
D5. Diminished interest in activities	HTQ13. Less interest in daily activities	–	NACM	NACM	AN
D6. Detachment	HTQ4. Feeling detached or withdrawn from people	–	NACM	NACM	AN
D7. Inability to feel positive emotions	HTQ5. Unable to show emotions	–	NACM	NACM	AN
E1. Irritability/anger	HTQ10. Feeling irritable or having outburst of anger	–	AR	DA	DA
E3. Hypervigilance	HTQ9. Feeling on guard	S	AR	AA	AA
E4. Exaggerated startle response	HTQ6. Feeling jumpy and easily startled	S	AR	AA	AA
E5. Difficulty in concentrating	HTQ7. Difficulty in concentrating	–	AR	DA	DA
E6. Sleep disturbance	HTQ8. Trouble sleeping	–	AR	DA	DA

HTQ, Harvard Trauma Questionnaire; RE, re-experiencing; A, avoidance; S, sense of threat; I, intrusions; NACM, negative alternations in cognition and mood; AR, arousal; AN, anhedonia; DA, dysphoric arousal; AA; anxious arousal.

a Elhai et al., 2011

b Liu et al., 2014.

DSM-5 diagnostic criteria for PTSD is met if participants endorsed at least one symptom of intrusion, one symptom of avoidance, two symptoms of NACM, and two symptoms of arousal. Symptom endorsement is indicated by item scores ≥3 as done originally in relation to the DSM-IV. Alternatively, the ICD-11 criteria is met if participants endorsed at least one symptom of each of the three clusters of re-experiencing, avoidance, and sense of threat; all indicated by scores ≥3 on the HTQ.

The Trauma Symptom Checklist (TSC; Briere & Runtz, 1989) contains 33 items; however, Elklit (1990) expanded the scale by adding two additional items. The TSC-35 contains seven subscales; depression, anxiety, dissociation, sleep-disturbances, somatization, interpersonal sensitivity, and aggression. For the purposes of the current study, we only considered the four subscales: depression, anxiety, dissociation, and aggression as these were deemed most appropriate for assessments of concurrent validity. Answers are rated on a four-point Likert scale (1=*never*, to 4=*often*). The TSC-35 has received support in multiple psychometric tests (Elklit, 1990). Reliability for each subscale within the full sample was satisfactory (*α*=0.70–0.93).

### Analysis

The dimensionality of the HTQ was investigated through the use of CFA techniques in Mplus version 7.00 (Muthén & Muthén, 2012) with robust maximum likelihood estimation (Yuan & Bentler, 2000). This method allowed parameters to be estimated using all available information and has been found to be superior to alternative methods such as listwise deletion (Schafer & Graham, 2002). Four models of the latent structure of PTSD were specified and estimated (Table 1).

Kline's (2011) suggestions for determination of good model fit were followed for the CFA analyses; *a chi-square-to-degrees of freedom* (χ^2^:df) ratio less than 3:1; *Comparative Fit Index* (CFI) and *Tucker Lewis Index* (TLI) values greater than 0.90 reflect acceptable model fit, and values greater than 0.95 reflect excellent fit; *root-mean-square error of approximation with 90% confidence intervals* (RMSEA 90% CI) and *standardized root-mean-square residual* (SRMR) values of 0.05 or less reflect excellent model fit, while values less than 0.10 reflect acceptable fit. Furthermore, the Akaike information criterion (AIC) can be used to evaluate alternative non-nested models, but this statistic cannot be used to compare the ICD-11 model with the DSM-5-based models as they comprise different number of variables. The CFI, RMSEA, and AIC all have explicit penalties for model.

Differences in diagnostic rates based on the two systems will be compared using the *z*-test, whereas Cohen's kappa coefficient (*k*) will be used to measure the level of agreement in diagnosis between the ICD-11 and the DSM-5.

## Results

### Model fit results

The CFA results indicated that the ICD-11 model provided an excellent representation of PTSD symptoms across six of the seven trauma samples, with the sole exception of the incest sample (Table 2). These model fit results provide strong support for the construct validity of the ICD-11 model of PTSD across a range of trauma types. The DSM-5 models failed to meet the threshold for acceptable fit in all seven samples. Interestingly, the DSM-5 and dysphoric arousal models performed similarly poorly across all samples. Of the three DSM-5-based models, the Anhedonia model provided the best fit although many of the observed factor correlations were extremely high (*r*'s >0.90) undermining the suitability of this model. Overall results indicate that the ICD-11 model provides an excellent fit of the data obtained from the different samples, and substantially better than any of the DSM-5 models. Additional CFA analyses were performed in Mplus version 7.00 with robust weighted least squares estimation (WLSMV) to further demonstrate the robustness of these results (Table 3). Of note, all other analyses were performed using the MLR estimator.

**Table 2:** Model fit statistics for the four PTSD models across all seven samples

Samples	*χ* ^2^	*df*	CFI	TLI	RMSEA (90% CI)	SRMR	AIC	BIC
1. Bereaved parents								
ICD-11	9	6	0.996	0.989	0.028 (0.000–0.063)	0.015	–	–
DSM-5	751*	164	0.850	0.826	0.073 (0.068–0.079)	0.057	26,874	27,171
Dysphoric arousal	681*	160	0.866	0.841	0.070 (0.065–0.075)	0.056	26,794	27,109
Anhedonia	442*	155	0.926	0.910	0.053 (0.047–0.059)	0.056	26,498	26,836
2. Whiplash victims								
ICD-11	12	6	0.997	0.992	0.025 (0.000–0.045)	0.010	–	–
DSM-5	1,596*	164	0.840	0.815	0.072 (0.069–0.076)	0.063	81,227	81,585
Dysphoric arousal	1,355*	160	0.866	0.841	0.067 (0.064–0.070)	0.059	80,975	81,354
Anhedonia	1,269*	155	0.875	0.847	0.066 (0.062–0.069)	0.053	80,858	81,264
3. Paraplegia sample								
ICD-11	2	6	1.000	1.054	0.000 (0.000–0.051)	0.014	–	–
DSM-5	295*	164	0.867	0.846	0.061 (0.049–0.072)	0.067	9,630	9,853
Dysphoric arousal	285*	160	0.874	0.850	0.060 (0.048–0.071)	0.066	9,623	9,852
Anhedonia	259*	155	0.895	0.871	0.056 (0.043–0.067)	0.062	9,598	9,860
4. Physical assault victims								
ICD-11	13	6	0.980	0.950	0.081 (0.019–0.139)	0.021	–	–
DSM-5	315*	164	0.895	0.878	0.070 (0.058–0.081)	0.059	9,626	9,840
Dysphoric arousal	290*	160	0.910	0.893	0.065 (0.053–0.077)	0.058	9,605	9,833
Anhedonia	273*	155	0.918	0.900	0.063 (0.051–0.075)	0.054	9,594	9,838
5. Incest victims								
ICD-11	48*	6	0.878	0.696	0.118 (0.089–0.150)	0.044	–	–
DSM-5	613*	164	0.765	0.728	0.074 (0.068–0.080)	0.068	26,087	26,366
Dysphoric arousal	610*	160	0.765	0.721	0.075 (0.069–0.081)	0.067	26,089	26,385
Anhedonia	490*	155	0.825	0.785	0.066 (0.059–0.072)	0.065	25,974	26,290
6. Rape victims								
ICD-11	11	6	0.987	0.967	0.054 (0.000–0.103)	0.021	–	–
DSM-5	468*	164	0.866	0.845	0.080 (0.071–0.088)	0.058	14,756	14,999
Dysphoric arousal	445*	160	0.874	0.850	0.078 (0.070–0.087)	0.056	14,741	14,998
Anhedonia	370*	155	0.905	0.884	0.069 (0.060–0.078)	0.058	14,666	14,943
7. Trauma patients								
ICD-11	7	6	0.993	0.983	0.035 (0.000–0.102)	0.027	–	–
DSM-5	359*	164	0.839	0.813	0.077 (0.066–0.087)	0.066	10,344	10,563
Dysphoric arousal	329*	160	0.860	0.834	0.072 (0.061–0.083)	0.063	10,318	10,550
Anhedonia	267*	155	0.907	0.886	0.060 (0.048–0.072)	0.060	10,261	10,509

*χ* ^2^, chi-square goodness of fit statistics; *df*, degrees of freedom; CFI, Comparative Fit Index; TLI, Tucker Lewis Index; RMSEA (90% CI), root-mean-square error of approximation with 90% confidence intervals; SRMR, standardized square root mean residual; AIC, Akaike information criterion; BIC, Bayesian information criterion; statistical significance

* *p*<0.0001.

**Table 3:** Model fit statistics for the four PTSD models across all seven samples using WLSMV estimator

Samples	*χ* ^2^	*df*	CFI	TLI	RMSEA (90% CI)	SRMR	AIC	BIC
1. Bereaved parents								
ICD-11	11	6	0.995	0.987	0.037 (0.000–0.069)	0.015	–	–
DSM-5	959*	164	0.847	0.823	0.085 (0.080–0.091)	0.057	26,874	27,171
Dysphoric arousal	871*	160	0.863	0.837	0.082 (0.076–0.087)	0.056	26,794	27,109
Anhedonia	566*	155	0.921	0.903	0.063 (0.058–0.069)	0.056	26,498	26,836
2. Whiplash victims								
ICD-11	12	6	0.997	0.993	0.026 (0.004–0.046)	0.010	–	–
DSM-5	1,736*	164	0.841	0.816	0.076 (0.073–0.079)	0.063	81,227	81,585
Dysphoric arousal	1,476*	160	0.867	0.842	0.070 (0.067–0.074)	0.059	80,975	81,354
Anhedonia	1,349*	155	0.880	0.852	0.068 (0.065–0.071)	0.053	80,858	81,264
3. Paraplegia sample								
ICD-11	3	6	1.000	1.024	0.000 (0.000–0.065)	0.014	–	–
DSM-5	364*	164	0.850	0.826	0.075 (0.065–0.085)	0.067	9,630	9,853
Dysphoric arousal	349*	160	0.858	0.832	0.074 (0.063–0.084)	0.066	9,623	9,852
Anhedonia	314*	155	0.881	0.854	0.062 (0.058–0.080)	0.062	9,598	9,860
4. Physical assault victims								
ICD-11	14	6	0.981	0.955	0.085 (0.027–0.143)	0.021	–	–
DSM-5	354*	164	0.887	0.869	0.078 (0.067–0.089)	0.059	9,626	9,840
Dysphoric arousal	326*	160	0.902	0.883	0.074 (0.062–0.085)	0.058	9,605	9,833
Anhedonia	304*	155	0.911	0.891	0.071 (0.059–0.083)	0.054	9,594	9,838
5. Incest victims								
ICD-11	42*	6	0.901	0.752	0.110 (0.080–0.142)	0.044	–	–
DSM-5	653*	164	0.766	0.729	0.077 (0.068–0.080)	0.068	26,087	26,366
Dysphoric arousal	647*	160	0.767	0.724	0.078 (0.072–0.084)	0.067	26,089	26,385
Anhedonia	521*	155	0.825	0.785	0.069 (0.062–0.075)	0.065	25,974	26,290
6. Rape victims								
ICD-11	12	6	0.987	0.966	0.059 (0.000–0.107)	0.021	–	–
DSM-5	504*	164	0.866	0.844	0.084 (0.076–0.093)	0.058	14,756	14,999
Dysphoric arousal	480*	160	0.873	0.850	0.083 (0.074–0.091)	0.056	14,741	14,998
Anhedonia	369*	155	0.905	0.883	0.073 (0.064–0.082)	0.058	14,666	14,943
7. Trauma patients								
ICD-11	8	6	0.992	0.979	0.041 (0.000–0.106)	0.027	–	–
DSM-5	381*	164	0.835	0.809	0.081 (0.070–0.091)	0.066	10,344	10,563
Dysphoric arousal	348*	160	0.857	0.831	0.076 (0.065–0.087)	0.063	10,318	10,550
Anhedonia	280*	155	0.905	0.883	0.063 (0.051–0.075)	0.060	10,261	10,509

*χ* ^2^, chi-square goodness of fit statistics; *df*, degrees of freedom; CFI, Comparative Fit Index; TLI, Tucker Lewis Index; RMSEA (90% CI), root-mean-square error of approximation with 90% confidence intervals; SRMR, standardized square root mean residual; AIC, Akaike information criterion; BIC, Bayesian information criterion; statistical significance

* *p*<0.0001.

The adequacy of the ICD-11 model was further indicated in relation to the robust parameter estimates and appropriate discrimination of latent factors. As detailed in Table 4, the ICD-11 model demonstrated satisfactory factor loadings within each sample. In each case, factor loadings were all positive, statistically significant, and greater than 0.40. Correlations between the three factors of the ICD-11 model were all statistically significant (*p*<0.001) and ranged between 0.46 and 0.85 across all samples.

**Table 4:** Standardized factor loadings (standard errors) for the six ICD-11 PTSD items across seven samples

Samples	HTQ2 (RE)	HTQ3 (RE)	HTQ11 (AV)	HTQ15 (AV)	HTQ6 (SOT)	HTQ9 (SOT)
Bereaved parents	0.64 (0.04)	0.70 (0.04)	0.81 (0.05)	0.62 (0.05)	0.77 (0.03)	0.82 (0.03)
Whiplash victims	0.75 (0.03)	0.65 (0.02)	0.71 (0.02)	0.65 (0.02)	0.67 (0.02)	0.76 (0.02)
Paraplegics	0.69 (0.10)	0.78 (0.09)	0.57 (0.09)	0.74 (0.10)	0.66 (0.08)	0.75 (0.09)
Physical assault victims	0.78 (0.05)	0.82 (0.04)	0.71 (0.06)	0.66 (0.07)	0.82 (0.04)	0.79 (0.04)
Incest victims	0.71 (0.06)	0.57 (0.06)	0.69 (0.08)	0.40 (0.06)	0.45 (0.07)	0.69 (0.09)
Rape victims	0.75 (0.05)	0.67 (0.05)	0.81 (0.06)	0.56 (0.06)	0.75 (0.05)	0.74 (0.04)
Trauma patients	0.61 (0.07)	0.68 (0.08)	0.71 (0.07)	0.53 (0.07)	0.80 (0.06)	0.67 (0.06)

All standardized factor loadings are statistically significant (*p*<0.001); HTQ, Harvard Trauma Questionnaire; RE, re-experiencing; AV, avoidance; SOT, sense of threat.

### Model invariance analyses

Given that the ICD-11 model was found to consistently provide a satisfactory representation of the data, it was feasible to merge all samples and conduct tests of model invariance for sex (males: *n*=1,069; females: *n*=2,663) using the ICD-11 model as the baseline. Following the procedures set forth by Sass (2011), we sought to determine if the ICD-11 model performs equally for males and females by testing for “strong factorial invariance.” This involves a series of steps: 1) assessing the fit of the ICD-11 model in males and females independently, 2) assessing configural invariance (ICD-11 model is tested simultaneously for males and females and estimated model parameters are allowed to differ across groups), 3) assessing metric invariance (factor loadings are constrained equal between males and females), and 4) assessing scalar invariance (intercepts are constrained equal). The configural model serves as a comparison model for the more parsimonious models in which the factor loadings, and intercepts, are constrained equal. Should these more parsimonious models provide equal or superior fit of the data, compared to the configural model, they are preferred on the grounds of parsimony and indicate that the ICD-11 model is invariant for sex.

All results are presented in Table 5, and as can be seen, the ICD-11 model fit the data very well for males and females independently. Metric and scalar invariance were supported based on the lower Bayesian information criterion (BIC) values relative to the configural model. These findings indicate that the ICD-11 performs equally for males and females.

**Table 5:** Test of sex invariance for the ICD-11 model of PTSD

Models	*χ* ^2^	*df*	CFI	TLI	RMSEA (90% CI)	SRMR	AIC	BIC
Males only	16.549*	6	0.993	0.982	0.041 (0.018–0.064)	0.014	–	–
Females only	35.640**	6	0.992	0.979	0.043 (0.033–0.057)	0.013	–	–
Configural invariance	363.874**	14	0.928	0.847	0.116 (0.106–0.126)	0.074	58,459	58,708
Metric invariance	372.784**	17	0.927	0.872	0.106 (0.097–0.115)	0.075	58,458	58,688
Scalar invariance	380.447**	21	0.927	0.895	0.096 (0.087–0.104)	0.077	58,458	58,663

*χ* ^2^, chi-square goodness of fit statistics; *df*, degrees of freedom; CFI, Comparative Fit Index; TLI, Tucker Lewis Index; RMSEA, root-mean-square error of approximation; SRMR, standardized square root mean residual; AIC, Akaike information criterion; BIC, Bayesian information criterion;

* *χ* ^2^ are statistically significant (*p*<0.01)

** *p*<0.0001.

#### Concurrent validity analyses

The concurrent validity of the ICD-11 model of PTSD was assessed by correlating the respective PTSD factors with the four subscales of the TSC among the full sample (Table 6). Each of the ICD-11 PTSD factors were moderately to strongly correlated with scores on depression, anxiety, dissociation, and aggression (*r*'s=0.42–0.92). Correlations between the DSM-5 factors of PTSD and the respective outcomes were also investigated in order to determine if the explanatory power of the ICD-11 model is reduced relative to the DSM-5 model due to the removal of a large number of symptoms. The correlations between the DSM-5 factors and the TSC subscales were of a similar magnitude (*r*'s=0.41–0.95). The DSM-5 arousal factor did appear to produce slightly stronger associations with depression, dissociation, and aggression, compared to the ICD-11 sense of threat factor. Considered in totality, these results indicate that associations with related outcomes are generally unaffected when using a far smaller set of symptoms in the ICD-11 model.

**Table 6:** Correlations (standard errors) between the latent ICD-11 and DSM-5 PTSD factors and the TSC subscales

Variables	Depression	Anxiety	Dissociation	Aggression
**Re-experiencing (ICD-11)**	**0.65 (0.02)**	**0.66 (0.02)**	**0.67 (0.02)**	**0.42 (0.02)**
Intrusions (DSM-5)	0.61 (0.02)	0.59 (0.02)	0.60 (0.02)	0.41 (0.02)
**Avoidance (ICD-11)**	**0.66 (0.02)**	**0.70 (0.02)**	**0.66 (0.02)**	**0.47 (0.02)**
Avoidance (DSM-5)	0.67 (0.02)	0.68 (0.02)	0.64 (0.02)	0.47 (0.02)
**Sense of Threat (ICD-11)**	**0.76 (0.01)**	**0.92 (0.01)**	**0.70 (0.02)**	**0.57 (0.02)**
Arousal (DSM-5)	0.91 (0.01)	0.95 (0.01)	0.88 (0.01)	0.76 (0.01)
NACM (DSM-5)	0.91 (0.01)	0.83 (0.01)	0.83 (0.01)	0.67 (0.01)

All correlations are statistically significant (*p*<0.001); *N*=3,746 for all correlations; TSC, Trauma Symptom Checklist; NACM, negative alterations in cognition and mood. ICD-11 factors in bold.

#### PTSD prevalence rates

Among the full sample, the PTSD prevalence rate was significantly higher for the DSM-5 than the ICD-11 (30.4 vs. 22.6%, *z*=8.88, *p*<0.001). Furthermore, the level of agreement between the two diagnostic systems was reasonable (82.4% agreement, *k*=0.581, *p*<0.001). The PTSD rates in each sample were as follows: bereaved parents (DSM-5=6.8%, ICD-11=5.4%, *z*=1.06, *p*=0.14), whiplash (DSM-5=31.4%, ICD-11=18.3%, *z=*8.54, *p*<0.001), paraplegics (DSM-5=5.2%, ICD-11=3.7%, *z*=0.77, *p*=0.22), physical assaults (DSM-5=31.3%, ICD-11=28.7%, *z=*0.50, *p=*0.29), incest victims (DSM-5=67%, ICD-11=52.1%, *z=*4.58, *p*<0.001), sexual assaults (DSM-5=43.7%, ICD-11=39.2%, *z*=1.07, *p*=0.14), and trauma patients (DSM-5=30.6%, ICD-11=33.8%, *z=*0.69, *p=*0.24). These results suggest a tendency for the DSM-5 to provide higher diagnostic rates compared to the ICD-11.

## Discussion

The current study sought to assess the validity of the newly proposed ICD-11 model of PTSD and offer a reasonably robust empirical comparison between the DSM-5 and ICD-11 conceptualizations of PTSD. Assessing the validity of both models is imperative as researchers and clinicians will soon be faced with the problem of deciding between two distinct methods of conceptualizing the same purported psychological experience; a decision which is likely to have substantial influence in informing understandings of the etiology and maintenance of PTSD, as well as its diagnosis and treatment (Elhai & Palmieri, 2011).

The CFA results showed that the ICD-11 model of PTSD provided an excellent representation of the structure of PTSD symptoms following exposure to a wide range of unique traumatic experiences. The only exception was with respect to the sample of incest survivors where model fit was unsatisfactory. The poorer fit of the ICD-11 model within this sample may be a reflection of the specific nature of the trauma. It is more common to observe complex-PTSD than PTSD among individuals who have been subjected to repeated sexual assault early in development (Cloitre et al., 2009); therefore, it may be the case that a large proportion of this sample was exhibiting signs of complex-PTSD which could explain the poorer model fit results. In contrast with the results for the ICD-11 model, the three DSM-5-based models of PTSD were all found to exhibit poor model fit across each of the trauma samples. Of the three DSM-5 models assessed, the Anhedonia model (Liu et al., 2014) was found to perform best across all trauma samples.

The poor fit observed for the DSM-5 models of PTSD may be due to the use of a DSM-IV measurement (the HTQ), whereas previous studies supporting the DSM-5 models achieved better model fit utilizing specific DSM-5 measurements (Liu et al., 2014; Miller et al., 2013) or other DSM-IV measurements with little or no modification in relation to the DSM-5 (Armour et al., 2014; Biehn et al., 2013; Contractor et al., 2014; Elhai et al., 2012). However, we argue that although the HTQ is a DSM-IV-based measure, it bears very close resemblance to the DSM-5 symptoms. At the very least, the HTQ appears to create a specific symptom profile that the DSM-5 models should be able to cover. Additionally, our measurement of PTSD symptoms did not separate the emotional and physiological arousal (criteria B4 and B5) and did not assess the DSM-5 E2 criterion of reckless or self-destructive behavior. However, these limitations are likely to be unimportant as research has shown that emotional and physiological reactivity are highly correlated and difficult to separate in clinical practice (Hansen et al., 2010), whereas reckless behavior appears not to be a good marker of PTSD as it does not load highly on its corresponding factor across various investigated models (Liu et al., 2014; Miller et al., 2013).

Results of the concurrent validity analyses provided further support for the ICD-11 model of PTSD. Not only did each of the factors correlate robustly with levels of depression, anxiety, dissociation, and aggression, the correlations were of a similar magnitude to those observed when the DSM-5 factors were correlated with the same outcomes. These results indicate that the explanatory power of the ICD-11 PTSD factors is largely unaffected by the removal of 14 symptoms. Thus, the much shortened ICD-11 model of PTSD provides a simpler and satisfactory description of posttraumatic stress responses without losing any explanatory power.

The simpler ICD-11 model has the benefit of simplifying clinical work given that clinicians need not worry about the thousands of combinations of symptom endorsement that can arise from the DSM-5 nosology (Maercker et al., 2013). This was also indicated by item response theory analyses conducted by Miller et al. (2013) on the DSM-5 PTSD measurement in the National Stressful Events Survey, which suggested that several items were providing largely redundant information especially within the criterion B symptoms.

Large differences in diagnostic rates between the DSM-5 criteria and the ICD-11 guidelines were observed among the full sample, with the former giving rise to significantly higher rates. Among the different trauma samples, the DSM-5 produced significantly higher diagnoses among the samples of incest survivors and whiplash victims. For the other five samples, there were no significant differences in diagnostic rates, although there was a trend for the DSM-5 to produce higher prevalence estimates. Extant results are generally consistent, therefore, with existing research regarding differences in prevalence between the two diagnostic nomenclatures (O'Donnell et al., 2014; Stein et al., 2014). Current and past results indicate that there may be a tendency for the DSM-5 to diagnose a larger number of people than the ICD-11; however, this appears to be somewhat dependent on the nature of the trauma experienced. Previous work from O'Donnell et al. (2014) suggested that the reduced diagnostic rates observed for the ICD-11 were related directly to the smaller number of possible re-experiencing and arousal symptoms. It was not possible in the current study to determine why the DSM-5 produced higher diagnostic rates; therefore, it is unclear if the DSM-5 is overinclusive and diagnosing individuals who are displaying normal levels of distress, or if the ICD-11 is too stringent and failing to capture people who are experiencing clinically meaningful psychological distress. Further research is clearly warranted to understand if there is indeed a consistent trend for the DSM-5 to diagnose a greater number of trauma survivors than the ICD-11 and to determine what factors might explain such a tendency.

The findings of the current study have several potential implications for clinical practice, research, and the general conceptualization of PTSD. First, results provide evidence to suggest that the latent structure of PTSD can be understood in a simpler manner than that which is outlined in the DSM-5. Thus, clinical work guided by the DSM-5 is potentially made more complicated than needed and may not be sufficiently targeting the right symptoms. As pointed out by Maercker et al. (2013), there are thousands of possible combinations of symptom endorsement that can arise from the DSM-5 nosology, making it difficult for clinicians to navigate within and treat these symptoms in a targeted and well-structured manner. This could potentially mean that the treatment becomes less efficient. The results further suggest that using the DSM-5 criteria rather than the proposed ICD-11 guidelines results in higher estimated PTSD prevalence rates; however, additional analyses are needed to determine whether this is a result of an overestimation of PTSD diagnoses by using the DSM-5 criteria or an underestimation of PTSD diagnoses by using the proposed ICD-11 criteria. It is important that diagnostic systems are precise, as they can be used to facilitate early treatment and prevention so that correct symptoms are targeted and the correct risk factors of developing posttraumatic stress symptoms are identified. Second, factor invariance testing on DSM-IV models indicated that there are sex differences on all factor structure parameters of different DSM-IV models (Armour et al., 2011). This is expected as women have twice the risk of developing PTSD following traumatic exposure as compared to males and are also more likely to develop chronic PTSD compared to males, thus pointing towards sex-specialized treatments (Armour et al., 2011). However, the ICD-11 PTSD model was found to perform equally well across sex and thus does not appear to require any specific sex-specialized treatment in regard to symptom configuration.

The current study had several limitations. First, PTSD symptoms in each sample were assessed using a self-report measure. It is possible that the latent structure of PTSD may differ depending on how it is assessed. Furthermore, despite a very close resemblance between the HTQ items and the DSM-5 PTSD criteria, potential bias connected to using a DSM-IV measurement rather than a DSM-5 measurement cannot be ruled out. Similarly, potential bias cannot be ruled out in relation to the lack of a separation of the B4 and B5 symptoms and a measurement of E2 in the current study. In a similar vein, the HTQ is not a precise and robust measurement of ICD-11 and we did not assess the presence of fear and horror. Future research with specific DSM-5 and ICD-11 self-report and clinically administered diagnostic interviews should be conducted. Second, the participants across the seven samples were all recruited from the Danish population and thus it is unknown whether the current results will generalize to other populations. It is important that future studies replicate these results in populations of children and adolescents, as well as non-Danish populations, as the results may have several important implications for research, theory, and clinical practice. Finally, all studies were cross-sectional and thus it was not possible to assess whether the ICD-11 model drives the course of PTSD and thus is stable across time. However, PTSD symptoms were assessed at different time points across the various trauma samples, suggesting that the ICD-11 model is temporally stable.

Despite its limitations, the current study is important as it adds substantially to the literature with regard to the construct validity of the newly proposed ICD-11 model of PTSD and is the first to simultaneously investigate the latent structure of PTSD with both DSM-5 models and the proposed ICD-11 model across the same samples of trauma-exposed individuals. Current results provide empirical support for the construct and concurrent validity of the ICD-11 model of PTSD which provides validation for the approach taken by the WHO to reduce the number of symptom indicators of PTSD. However, far greater research will be required to determine if the ICD-11 proposals are indeed accurate and clinically useful. We believe it to be critical to continue to find novel ways of assessing the different models of PTSD outlined in the DSM-5 and the ICD-11 to better determine the most accurate symptom profile of PTSD. The availability of two diagnostic nomenclatures that present widely differing symptom profiles has the potential to give rise to a situation where important data that are gathered using distinct models cannot be reconciled. Important information could therefore be lost and ultimately the capacity of mental health professionals to help those who have experienced trauma diminished.
